# Revision Carpal Tunnel Release Following Endoscopic Compared With Open Decompression

**DOI:** 10.1001/jamanetworkopen.2023.52660

**Published:** 2024-01-12

**Authors:** Peter C. Ferrin, Bergen K. Sather, Kelsi Krakauer, Timothy P. Schweitzer, Angelo B. Lipira, Ravi F. Sood

**Affiliations:** 1 Department of Surgery, Oregon Health & Science University, Portland; 2 Department of Surgery, Virginia Mason Medical Center, Seattle, Washington; 3 Division of Plastic and Reconstructive Surgery, Department of Surgery, Stanford University, Palo Alto, California; 4 VA Puget Sound Health Care System–American Lake Division, Tacoma, Washington; 5 Division of Plastic and Reconstructive Surgery, Department of Surgery, Oregon Health & Science University, Portland; 6 Operative Care Division, Portland VA Medical Center, Portland, Oregon; 7 Division of Plastic and Reconstructive Surgery, Department of Surgery, University of California Davis, Sacramento

## Abstract

**Importance:**

Carpal tunnel release (CTR) technique may influence the likelihood of revision surgery. Prior studies of revision CTR following endoscopic CTR (ECTR) compared with open CTR (OCTR) have been limited by sample size and duration of follow-up.

**Objective:**

To estimate the incidence of revision CTR following ECTR compared with OCTR in a national cohort.

**Design, Setting, and Participants:**

This retrospective cohort study used data from the US Veterans Health Administration. Participants included all adults (age ≥18 years) undergoing at least 1 outpatient CTR from October 1, 1999, to May 20, 2021. Data were analyzed from May 21, 2021, to November 27, 2023.

**Exposure:**

Index CTR technique.

**Main Outcomes and Measures:**

The primary outcome was time to revision CTR, defined as repeat ipsilateral CTR during the study period. Secondary outcomes were indications for revision, findings during revision, and additional procedures performed during revision.

**Results:**

Among 134 851 wrists from 103 455 patients (92 510 [89.4%] male; median [IQR] age, 62 [53-70] years) undergoing at least 1 CTR, 1809 wrists underwent at least 1 revision at a median (IQR) of 2.5 (1.0-3.8) years. In competing-risks analysis, the cumulative incidence of revision was 1.06% (95% CI, 0.99%-1.12%) at 5 years and 1.59% (95% CI, 1.51%-1.67%) at 10 years. ECTR was associated with increased hazard of revision CTR compared with OCTR (adjusted hazard ratio [aHR], 1.56; 95% CI, 1.34-1.81; *P* < .001). The risk difference for revision CTR associated with ECTR compared with OCTR was 0.57% (95% CI, 0.31%-0.84%) at 5 years (number needed to harm, 176) and 0.72% (95% CI, 0.36%-1.07%) at 10 years (number needed to harm, 139). Regardless of index CTR technique, the most common indication for revision was symptom recurrence (1062 wrists [58.7%]). A reconstituted transverse carpal ligament (TCL) was more common after ECTR compared with OCTR, whereas scarring of the overlying tissues and of the median nerve itself were more common following OCTR. Incomplete transverse-carpal-ligament release was observed in 251 of the wrists undergoing revision CTR (13.94%) and was more common among revisions following ECTR (odds ratio, 1.62; 95% CI, 1.11-2.37; *P* = .01).

**Conclusions and Relevance:**

In this cohort study of revision CTR in the Veterans Health Administration, ECTR was associated with increased risk of revision compared with OCTR, but the absolute risk was low regardless of technique. Intraoperative findings at revision varied significantly according to index CTR technique.

## Key Points

**Question:**

Does the incidence of revision carpal tunnel release (CTR) vary in association with index CTR technique?

**Findings:**

In this cohort study of 134 851 wrists from 103 455 patients undergoing CTR in the Veterans Health Administration, endoscopic CTR was associated with a significantly higher hazard of revision; however, the incidence of revision was low regardless of index CTR technique, with a risk difference of 0.72% at 10 years for endoscopic CTR.

**Meaning:**

These findings suggest that although endoscopic CTR was associated with a higher incidence of revision compared to open CTR, the absolute risk was low regardless of technique.

## Teaser

This cohort study assesses the incidence of revision carpal runnel release after endoscopic vs open carpal tunnel release among patients in the Veterans Health Administration.

## Introduction

Carpal tunnel release (CTR) is among the most common operations performed in the US and is generally safe and effective.^1,2,3^ However, revision surgery has been estimated to occur in 1% to 5% of patients^2,4,5^ for indications including failure to relieve the presenting symptoms, symptom recurrence following a symptom-free interval, or development of new symptoms.^6^ Although outcomes data are lacking, revision CTR has been associated with unsatisfactory results, including persistence of symptoms and decreased patient-reported pain and satisfaction scores,^7,8,9^ and there is accordingly interest in identifying factors leading to revision.

One such factor of interest is CTR technique. Although open CTR (OCTR) remains most common, endoscopic CTR (ECTR) has gained popularity in the US^10,11,12^ since its introduction more than 30 years ago.^13,14^ Controversy persists regarding the risks and benefits of ECTR vs OCTR, with one concern being the potential for incomplete release of the transverse carpal ligament (TCL) during ECTR.^9,15,16,17^ Although numerous studies have assessed the association between surgical technique and risk of revision CTR, results have been inconsistent and limited by paucity of patients undergoing revision CTR and relatively short follow-up.^1,4,5,18,19,20,21^ Given the infrequency of revision CTR and unknown natural history of symptom recurrence, large sample size and long follow-up are essential to properly study this outcome.^22^

Hence, our primary objective was to estimate the incidence of revision CTR associated with ECTR relative to OCTR in a large national cohort. Our secondary objective was to compare indications for and procedural details of revision CTR following ECTR and OCTR.

## Methods

### Study Design, Population, and Setting

This cohort study was approved by the Portland VA Medical Center institutional review board. A waiver of informed consent was granted because this study was a retrospective analysis of an existing database. This study is reported following the Strengthening the Reporting of Observational Studies in Epidemiology (STROBE) reporting guideline.

Participants included all adults (age ≥18 years) undergoing at least 1 outpatient CTR at any of the 130 Veterans Health Administration (VHA) facilities in the US from October 1, 1999, to May 20, 2021. Patients were identified in the VHA Corporate Data Warehouse based on a *Current Procedural Terminology* (CPT) code for OCTR (64721) or ECTR (29848) associated with a diagnosis code from *International Classification of Diseases, Ninth Revision (ICD-9)* (code 354.0) or *International Statistical Classification of Diseases and Related Health Problems, Tenth Revision (ICD-10) *(code G56.00-G56.03) for carpal tunnel syndrome.

### Exposures and Outcomes

The primary exposure was surgical technique. The primary outcome was time to repeat ipsilateral CTR. Procedure laterality was determined by CPT modifier codes when present and otherwise by natural language processing of operative records, with an estimated accuracy of 99.5% (eMethods in Supplement 1). Procedures for which laterality could not be determined in this manner (<1%) were excluded, and laterality of index and revision CTRs was confirmed by manual electronic medical record review for all revision CTRs (eFigure in Supplement 1). Covariates included demographic variables and comorbidities that might confound the association between operative technique and need for revision,^5^ the latter defined by at least 1 *ICD-9* or *ICD-10* diagnosis code for the condition preceding the index CTR. Race and ethnicity were self-reported and categorized as Black, White, or other (eg, American Indian or Alaska Native, Asian, Hawaiian or Pacific Islander, multiple races, or unknown) race and Hispanic, non-Hispanic, or unknown ethnicity. Race was considered in the analysis as a potential confounder, as race has previously been shown to be independently associated with treatment modality in carpal tunnel syndrome.^23^ In addition, race may be associated with genetic factors that could influence the rate of revision surgery, for instance by affecting scarring phenotype or risk of amyloidosis.^24^

In the secondary analysis, outcomes of interest pertained to the revision CTR, including indications, intraoperative findings, and additional procedures performed. These details were ascertained via manual review of the operative reports and perioperative clinic notes from the electronic medical record. Indication for reoperation was categorized according to an established framework,^6^ with *recurrent symptoms* defined as relief of symptoms for any period followed by the redevelopment of the same symptoms. *Incomplete TCL release* was designated when specifically documented by the surgeon performing the revision CTR. *Reconstituted TCL* was designated when specifically documented by the reoperative surgeon or when division of the TCL was described during the revision CTR without mention of it appearing to have been incompletely released.

### Statistical Analysis

We used time-to-event analysis to account for differential follow-up and death. As each patient could undergo CTR on either or both wrists, each wrist was analyzed separately for recurrence. Study time began for a given wrist at the time of the first CTR on that side and continued until repeat ipsilateral CTR was performed or the patient died. Death without revision CTR was analyzed as a competing event, and wrists without either event were censored at the latest follow-up in the VHA (no later than May 20, 2021).

In the primary analysis, cumulative incidence of revision CTR was estimated using the cumulative incidence function. Fine-Gray subdistribution hazard regression was used to estimate crude hazard ratios (HRs) and adjusted subdistribution HRs (aHRs) associated with revision CTR following ECTR compared with OCTR. In the secondary analysis, we used bivariate logistic (for dichotomous dependent variables) or linear (for continuous dependent variables) regression to test for associations of index CTR technique with operative indications, findings, and associated procedures performed. In primary and secondary analyses, all regression models were fit with cluster-robust standard errors accounting for correlation between wrists of the same patient.^25^ Statistical inference was based on null-hypothesis testing of regression coefficients, with 2-sided α = .05. All analyses were performed using R version 4.1.2 (R Project for Statistical Computing) in the VA Informatics and Computing Infrastructure. Data were analyzed from May 21, 2021, to November 27, 2023.

## Results

Among 103 455 patients undergoing at least 1 CTR in the VHA during the nearly 22-year study period, the median (IQR) age was 62 (53 to 70) years and 92 510 patients (89.4%) were men. The cohort included 5081 Hispanic patients (4.9%) and 94 179 non-Hispanic patients (91.0%), and there were 12 402 Black or African American patients (12.0%), 80 881 White patients (78.2%), and 10 172 patients (9.8%) who identified as another race. Approximately one-third of patients underwent bilateral CTRs, for a total of 134 851 wrists analyzed (Table 1; eFigure in Supplement 1). The index CTR was endoscopic in 10 226 wrists (7.6%), with ECTR more common among younger patients, women, and patients undergoing bilateral CTRs and less common among patients with diabetes or rheumatoid arthritis (Table 1). Death occurred in 22 764 patients (22.0%). Median (IQR) follow-up from index CTR to either the first revision, death, or last follow-up in the VHA was 6.5 (3.5 to 10.4) years and was similar for wrists undergoing index ECTR (median [IQR], 6.4 [3.1 to 10.5] years) and OCTR (median [IQR], 6.5 [3.5 to 7.3] years).

**Table 1.** Characteristics of Patients and Wrists Undergoing at Least 1 CTR During the Study Period

Characteristic	No. (%)			*P* value^a^
	Patients (n = 103 455), No. %	Wrists by index CTR technique		
		Open (n = 124 625)	Endoscopic (n = 10 226)	
Age, median (IQR), y	62 (53-70)	62 (53-70)	60 (51-68)	<.001
Sex				
Female	10 945 (10.6)	13 363 (10.7)	1266 (12.4)	<.001
Male	92 510 (89.4)	111 262 (89.3)	8960 (87.6)	
Ethnicity				
Hispanic	5081 (4.9)	6181 (5.0)	416 (4.1)	<.001
Non-Hispanic	94 179 (91.0)	113 668 (91.2)	9274 (90.7)	
Unknown	4197 (4.1)	4776 (3.8)	536 (5.2)	
Race				
Black or African American	12 402 (12.0)	14 279 (11.5)	1185 (11.6)	<.001
White	80 881 (78.2)	98 462 (79.0)	7825 (76.5)	
Other^b^	10 172 (9.8)	11 884 (9.5)	1216 (11.9)	
Comorbidities^c^				
Amyloidosis	62 (0.1)	80 (0.1)	3 (<0.1)	.19
Diabetes	36 028 (34.8)	43 097 (34.6)	3352 (32.8)	.002
Obesity	47 256 (45.7)	57 753 (46.3)	4772 (46.7)	.59
Rheumatoid arthritis	3445 (3.3)	4200 (3.4)	283 (2.8)	.005
Smoking	39 503 (38.2)	47 414 (38.0)	3800 (37.2)	.13
Carpal tunnel syndrome laterality^d^				
Unilateral	72 053 (69.6)	67 354 (54.0)	4699 (46.0)	<.001
Bilateral	31 399 (30.4)	57 271 (46.0)	5527 (54.0)	
Index CTR laterality				
Unilateral	101 246 (97.9)	121 437 (97.4)	8999 (88.0)	<.001
Bilateral	2209 (2.1)	3188 (2.6)	1227 (12.0)	

Abbreviation: CTR, carpal tunnel release.

^a^ Based on linear or logistic regression as appropriate, accounting for correlation between wrists from the same patient.

^b^ Including individuals self-identifying as American Indian or Alaska Native, Asian, Hawaiian or Pacific Islander, multiple races, or unknown.

^c^ For patients, comorbidities shown were as of the earliest CTR performed on either side (for those undergoing ≥1 CTR on each side). For wrists, comorbidities shown were as of the time of the index CTR on that side.

^d^ Defined as unilateral for patients undergoing 1 or more CTRs on 1 wrist only and bilateral for those undergoing at least 1 CTR on each wrist.

Among 134 851 wrists undergoing at least 1 CTR, 1809 underwent at least 1 revision in the VHA, at a median (IQR) interval of 2.5 (1.0 to 3.8) years. Forty wrists went on to second revisions, performed at a median (IQR) of 2.2 (1.0 to 4.7) years after the first revision and 6.7 (2.8 to 9.6) years from the index CTR; all ensuing analyses are based on the first revisions only. Of these, almost all revisions (1781 revisions [98.5%]) were performed as OCTR, with 28 first revisions (1.5%) performed at ECTR. Accounting for variable length of follow-up and the competing risk of death, the cumulative incidence of first revision CTR was 0.34% at 1 year, 1.06% at 5 years, 1.59% at 10 years, and 1.97% at 15 years (Figure, A, and Table 2). The risk difference for revision CTR associated with ECTR compared with OCTR was 0.57% (95% CI, 0.31%-0.84%) at 5 years (number needed to harm, 176) and 0.72% (95% CI, 0.36%-1.07%) at 10 years (number needed to harm, 139) (Table 2). Of 10 226 wrists undergoing index ECTR, 198 underwent revision at median (IQR) of 1.9 (0.8 to 5.4) years, compared with 1611 of 124 625 wrists that underwent index OCTR, at a median (IQR) of 2.6 (1.0 to 5.7) years. In unadjusted competing-risks analysis, wrists undergoing ECTR had a significantly higher cumulative incidence of revision compared with those undergoing OCTR (HR, 1.58; 95% CI, 1.36 to 1.83; *P* < .001) (Figure, C, and Table 2). In multivariable analysis, ECTR remained associated with a significantly higher cumulative incidence of revision (aHR, 1.56; 95% CI, 1.34 to 1.81; *P* < .001), as did male sex and bilateral carpal tunnel syndrome (Table 3). In a second model including the same covariates as well as an interaction term between patient sex and index CTR technique, the interaction term was not significant (coefficient estimate −0.25; 95% CI, −0.77 to 0.25; *P* = .33), indicating that the association between index CTR technique and hazard of revision did not depend on sex (Figure, D).

**Figure.**
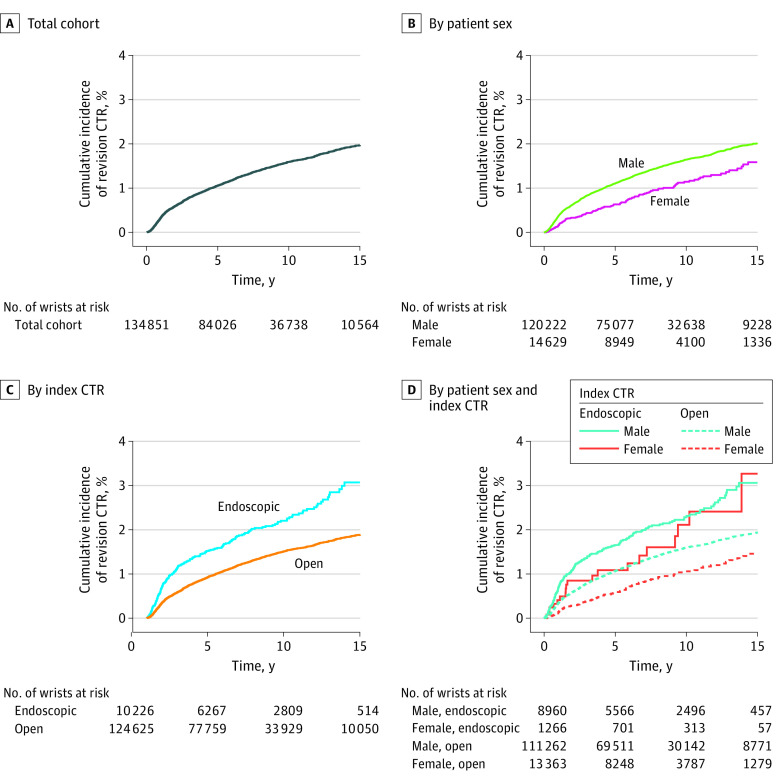
Cumulative Incidence Function for Revision Carpal Tunnel Release (CTR) in the Veterans Health Administration From 1999-2021 Death was treated as a competing risk.

**Table 2.** Risk Difference and Number Needed to Harm for Revision CTR Following ECTR vs OCTR

Time, y	Cumulative incidence of revisions (95% CI)^a^			Risk difference (95% CI)^b^	No. needed to harm
	Overall (n = 134 851)	OCTR (n = 124 625)	ECTR (n = 10 226)		
1	0.34 (0.31-0.37)	0.32 (0.29-0.35)	0.65 (0.50-0.81)	0.34 (0.18-0.50)	295
5	1.06 (0.99-1.12)	1.01 (0.95-1.08)	1.59 (1.33-1.84)	0.57 (1.31-0.84)	176
10	1.59 (1.51-1.67)	1.54 (1.45-1.62)	2.25 (1.91-2.59)	0.72 (0.36-1.07)	139
15	1.97 (1.86-2.07)	1.88 (1.77-2.00)	3.07 (2.54-3.60)	1.19 (0.63-1.74)	85

Abbreviations: CTR, carpal tunnel release; ECTR, endoscopic carpal tunnel release; OCTR, open carpal tunnel release.

^a^ Based on cumulative incidence function (Figure), accounting for differential follow-up and death during the study period.

^b^ Calculated as cumulative incidence of revision CTR following ECTR – cumulative incidence of revision CTR following OCTR; value may not correspond directly to difference between adjacent 2 columns due to rounding.

**Table 3.** Multivariable Competing Risks Regression Model of Revision CTR^a^

Characteristic	aHR (95% CI)	*P* value
Age, per 1-y increase	1.00 (1.00-1.00)	.79
Sex		
Female	1 [Reference]	NA
Male	1.51 (1.25-1.83)	<.001
Race		
Black or African American	1.13 (0.97-1.31)	.11
White	1 [Reference]	NA
Other^b^	0.83 (0.70-0.99)	.04
Comorbidities^c^		
Diabetes	1.12 (1.01-1.24)	.03
Obesity	0.96 (0.87-1.06)	.43
Rheumatoid arthritis	1.11 (0.86-1.42)	.43
Smoking	0.91 (0.82-1.01)	.07
Carpal tunnel syndrome laterality^d^		
Unilateral	1 [Reference]	NA
Bilateral	1.33 (1.21-1.46)	<.001
Year of index CTR, per 1-y increase	0.98 (0.97-0.99)	<.001
Index CTR technique		
Open	1 [Reference]	NA
Endoscopic	1.56 (1.34-1.81)	<.001

Abbreviations: aHR, adjusted subdistribution hazard ratio; CTR, carpal tunnel release; NA, not applicable.

^a^ Based on multivariable Fine-Gray subdistribution hazard regression including all variables listed, analyzing death as a competing event, and accounting for correlation between wrists from the same patient.

^b^ Including those self-identifying as Asian, Hawaiian or Pacific Islander, American Indian or Alaska Native, multiple races, or unknown.

^c^ Documented as of index CTR date.

^d^ Defined as unilateral for patients undergoing one or more CTRs on one wrist only and bilateral for those undergoing at least one CTR on each wrist.

Regardless of index CTR technique, the most common indication for revision was recurrence of symptoms (1062 wrists [58.7%) (Table 4), with revision performed at a median (IQR) of 4.1 (1.8 to 7.1) years. Revision for persistent symptoms was the second most common indication (718 wrists [39.7%]), performed at a median (IQR) of 1.2 (0.7 to 2.7) years, a significantly shorter interval than revision for recurrent symptoms (mean difference, −2.8 years; 95% CI, −3.1 to −2.5 years; *P* < .001). Revision for persistent symptoms was more common after ECTR (93 wrists [47.0%]) compared with OCTR (625 wrists [39.8%]) (adjusted *P* = .06). Other indications for revision were rare (Table 4).

**Table 4.** Details of Revision CTR Procedures Following Endoscopic Compared to Open Index CTR

Characteristic	Wrists, No. (%)			OR (95% CI)	*P* value^a^
	Overall (n = 1809)	Index CTR technique			
		Open (n = 1611)	Endoscopic (n = 198)		
Revision CTR surgeon					
Different from index CTR surgeon	1266 (70.0)	1.137 (70.6)	129 (65.2)	NA	.24
Same as index CTR surgeon	540 (29.8)	471 (29.2)	69 (34.8)	NA	
Undetermined	3 (0.2)	3 (0.2)	0	NA	
Primary indication for revision					
Recurrent symptoms	1062 (58.7)	961 (59.7)	101 (51.0)	0.71 (0.52-0.95)	.04^b^
Persistent symptoms					
Any	718 (39.7)	625 (38.8)	93 (47.0)	1.40 (1.04-1.88)	.06^b^
Consistent	666 (36.8)	577 (35.8)	89 (44.9)	NA	NA
Worsening	52 (2.9)	48 (3.0)	4 (2.0)	NA	NA
New symptoms	7 (0.4)	6 (0.4)	1 (0.5)	NA	NA
Infection	3 (0.2)	3 (0.2)	0	NA	NA
Other^c^	5 (0.3)	4 (0.2)	1 (0.5)	NA	NA
Undetermined	14 (0.8)	12 (0.7)	2 (1.0)	NA	NA
Intra-operative findings at revision					
Reconstituted TCL	960 (53.1)	838 (52.0)	122 (61.6)	1.48 (1.09-2.01)	.01
Incomplete release of TCL, portion					
Any	251 (13.9)	212 (13.2)	39 (19.7)	1.62 (1.11-2.37)	.01
Portion of TCL not released^d^					.01
Proximal only	85 (33.9)	78 (36.8)	7 (17.9)	NA	NA
Distal only	110 (43.8)	92 (43.4)	18 (46.2)	NA	NA
Proximal and distal	21 (8.4)	18 (8.5)	3 (7.7)	NA	NA
Central	3 (1.2)	1 (0.5)	2 (5.1)	NA	NA
Unspecified	32 (12.7)	23 (10.8)	9 (23.1)	NA	NA
Overlying soft-tissue scarring	864 (47.8)	801 (49.7)	63 (31.8)	0.47 (0.34-0.65)	<.001
Nerve scarring	816 (45.1)	744 (46.2)	72 (36.4)	0.67 (0.49-0.91)	.01
Nerve injury	18 (1.0)	16 (1.0)	2 (1.0)	1.02 (0.23-4.46)	.98
Synovitis	149 (8.2)	135 (8.4)	14 (7.1)	0.83 (0.47-1.48)	.53
Mass	18 (1.0)	17 (1.1)	1 (0.5)	NA	NA
Other	36 (2.0)	31 (1.9)	5 (2.5)	NA	NA
Undetermined	18 (1.0)	16 (1.0)	2 (1.0)	NA	NA
Additional procedures performed during revision					
Neurolysis^e^	735 (40.6)	674 (41.8)	61 (30.8)	0.70 (0.52-0.95)	.02
Flap	182 (10.1)	171 (10.6)	11 (5.6)	0.50 (0.26-0.93)	.03
Hypothenar fat pad	165 (9.1)	156 (9.7)	9 (4.5)	NA	NA
Other	17 (0.9)	15 (0.9)	2 (1.0)	NA	NA
Synovectomy	159 (8.8)	138 (8.6)	21 (10.6)	1.27 (0.78-2.06)	.34
Nerve wrap	121 (6.7)	105 (6.5)	16 (8.1)	1.26 (0.73-2.19)	.49
Corticosteroid injection into the carpal tunnel	25 (1.4)	21 (1.3)	4 (2.0)	NA	NA
Median nerve decompression in the forearm	19 (1.1)	19 (1.2)	0	NA	NA
Mass excision from carpal tunnel	15 (0.8)	13 (0.8)	2 (1.0)	NA	NA
Nerve repair or graft	14 (0.8)	12 (0.7)	2 (1.0)	1.36 (0.30-6.12)	.69
Tenolysis	8 (0.4)	7 (0.4)	1 (0.5)	NA	NA
Tenosynovial biopsy	5 (0.3)	4 (0.2)	1 (0.5)	NA	NA
Fat grafting or acellular dermal matrix	5 (0.3)	5 (0.3)	0	NA	NA
Opponensplasty	4 (0.2)	4 (0.2)	0	NA	NA
Irrigation and debridement	2 (0.1)	2 (0.1)	0	NA	NA

Abbreviations: CTR, carpal tunnel release; NA, not applicable; TCL, transverse carpal ligament.

^a^ Based on logistic regression, accounting for correlation between wrists from the same patient.

^b^ Adjusted for 2 comparisons by Bonferroni correction.

^c^ Including flexor-tendon procedures (2 wrists), Guyon canal release (2 wrists), and ganglion excision (1 wrist).

^d^ Percentage denominators are the 251 wrists found to have an incompletely released TCL during revision CTR.

^e^ Neurolysis was defined by the release of external or internal nerve scar tissue to any extent with or without an operating microscope.

The most common findings during revision reflected scar-tissue formation, with TCL reconstitution, overlying soft-tissue scarring, or nerve scarring described in approximately one-half of all wrists (Table 4). A reconstituted TCL was more common after ECTR compared with OCTR, whereas scarring of the overlying soft tissues and of the median nerve itself were more common following OCTR (Table 4). Incomplete TCL release was observed in 251 of the wrists undergoing revision CTR (13.94%) and was more common among revisions following ECTR (odds ratio [OR], 1.62; 95% CI, 1.11 to 2.37; *P* = .01). In wrists with incomplete TCL release, the distal portion was intact most commonly regardless of index CTR technique. However, an intact proximal TCL was seen more commonly after OCTR and intact distal portion was seen more commonly after ECTR (Table 4). The likelihood of documented incomplete TCL release did not depend on whether the revision was performed by a different surgeon (1266 wrists [70.0%]) from the index surgeon (OR, 1.00; 95% CI, 0.75 to 1.34; *P* = .99).

Findings during revision also varied according to surgical indication. Incomplete TCL release was more commonly documented during revision for symptom persistence (176 of 718 wrists [24.5%]) compared with recurrence (71 of 1062 wrists [6.7%]; OR 4.53; 95% CI, 3.36 to 6.11; *P* < .001). Median-nerve scarring was noted in 354 of 718 wrists (49.3%) revised for symptom persistence, compared with 451 of 1062 wrists (42.5%) revised for recurrence (OR, 1.32; 95% CI, 1.09 to 1.60, *P* = .005). In contrast, TCL reconstitution was seen more frequently in revisions for recurrent symptoms (633 wrists [59.6%]) compared with revisions for persistent symptoms (316 wrists [44.0%]; OR, 1.88; 95% CI, 1.55 to 2.28; *P* < .001). Overlying soft-tissue scarring was equally common among wrists revised for recurrent (511 wrists [48.1%]) and persistent (345 wrists [48.1%]) symptoms (OR, 1.00; 95% CI, 0.83 to 1.21; *P* = .98).

Related additional procedures were performed during approximately one-half of revision CTRs (916 of 1809 revision CTRs [50.6%]). Undergoing at least 1 additional procedure was less common following ECTR (76 of 198 revision CTRs [38.4%]) compared with OCTR (840 of 1611 revision CTRs [52.1%]; OR, 0.57; 95% CI, 0.42 to 0.78; *P* < .001), although the number of additional procedures was similar between groups (mean [SD], 0.60 [0.91] additional procedures after ECTR vs 0.73 [0.86] additional procedures after OCTR; mean difference, −0.13; 95% CI, −0.26 to 0.01; *P* = .06). The most common additional procedures were neurolysis, flaps, synovectomy, and nerve wraps; neurolysis and flaps (predominantly hypothenar fat pad flaps) were more common during revision of OCTRs compared with ECTRs, with the frequency of synovectomy and nerve wraps comparable (Table 4). Injury to the median nerve or 1 of its branches was identified during 18 revision CTRs (1.0%), with an equal frequency following ECTR compared with OCTR (Table 4).

## Discussion

This cohort study analyzed data from the largest integrated health care system in the US to obtain robust estimates of the incidence of revision CTR overall and following ECTR compared with OCTR during a nearly 22-year period. To our knowledge, we report the largest study on this topic to date, providing important information to help guide surgeons and patients in their selection of CTR technique. Although revision CTR is uncommon, delineating its incidence and risk factors is important for public health, considering that CTR is among the most common operations in the US^3^ and that outcomes of revision CTR are suboptimal.^7,8,9^ With recent studies reporting an increased risk of revision CTR following ECTR,^4,5,20^ precise characterization of this association is needed.

In our competing-risks analysis, we estimated a cumulative incidence of revision CTR of 0.34% at 1 year, 1.06% at 5 years, 1.59% at 10 years, and 1.97% at 15 years. By comparison, in a large single-institution retrospective cohort study of revision CTR, Westenberg et al^5^ reported a crude incidence of revisions CTR in 1.3% of wrists, similar to the crude incidence we observed (1.3%). However, the shorter duration of follow-up (4.8 years) and interval to revision surgery (1.2 years) in the study by Westenberg et al^5^ compared with ours (6.5 years and 2.5 years, respectively) indicate a higher incidence rate of revision CTR in their analysis. In a retrospective analysis of the national PearlDiver database, Wessel et al^4^ reported an even higher crude incidence of revision CTR (4.8%) at a mean interval of 129 days (0.35 years),^4^ consistent with their exclusion of patients lost to follow-up and limitation of their analysis to revisions performed within 1 year. A 2023 single-center cohort study by Carroll et al^20^ used a similar design and found a revision CTR incidence of 1.0% at a mean interval of 143 days (0.39 years). In contrast to these results, our cumulative incidence estimates are based on a much larger national cohort with more revision CTRs, account for differential patient follow-up, and reflect revisions performed for both persistent and recurrent symptoms, with recurrent symptoms generally occurring after a longer interval.

We found ECTR to be associated with a higher hazard of revision CTR compared to OCTR. This observation is similar to results from prior retrospective cohort studies, including Westenberg et al^5^ Wessel et al,^4^ and Carroll et al.^20^. Although randomized trials of ECTR vs OCTR have not shown a significant association between CTR technique and recurrence or reoperation, these studies were underpowered to assess these rare and delayed secondary outcomes, even when analyzed in aggregate.^1,18,19^ Our study adds evidence that ECTR is associated with significantly increased rate of revision. However, it must be emphasized that the absolute risk of revision CTR was low regardless of the index CTR technique. For instance, the cumulative incidence of revision CTR after ECTR compared with OCTR was 2.25% vs 1.54% at 10 years, corresponding to an absolute risk difference of only 0.72% and number needed to harm of 139. While statistically significant, a difference of this magnitude may not be clinically important, especially considering that surgeons might have a lower threshold to recommend revision CTR following ECTR compared with OCTR due to heightened suspicion of incomplete TCL release and possibility of nerve injury, in particular when the index CTR was performed by a different surgeon. Therefore, our results suggest that differential risk of revision CTR may not be an important reason to choose one CTR technique over another.

In our cohort, symptom recurrence was the most common indication for revision CTR (59% of revisions) followed by symptom persistence (40% of revisions). Although prior studies have reported symptom persistence as the most common indication,^5,6,8,11,15^ they were based on smaller cohorts with shorter follow-up, supporting the call for longer-term studies.^22^ We found that revision for recurrent symptoms occurred at a significantly longer median interval of 4.1 years, compared with 1.2 years for persistent symptoms, and our data indicate that reoperation for recurrence of symptoms was more common than previously recognized.

We report a higher prevalence of incomplete TCL release and reconstituted TCL and lower prevalence of nerve- and overlying soft-tissue scarring at revision CTR following ECTR compared with OCTR, with neurolysis and flaps more commonly performed during revision after OCTR. Based on these findings, we speculate that different patterns of soft-tissue healing may result from ECTR compared with OCTR, corresponding to differential propensity for recurrent symptoms even many years later. Although we did not assess outcomes of revision CTR, refractory or recurrent symptoms secondary to nerve scarring may require different treatment and carry a different prognosis for relief following revision surgery compared with ongoing or recurrent nerve compression. Accordingly, some prior studies have reported a greater likelihood of symptomatic relief following revision CTR where incomplete release was treated.^17,26,27^ Further studies on outcomes of revision CTR surgery are required to test this hypothesis.

The distal TCL was the portion most commonly intact in cases of incomplete TCL release following ECTR or OCTR. Although the potential for surgical failure due to incomplete release of the proximal TCL or antebrachial fascia during OCTR is well described, along with techniques aimed at preventing it,^28,29^ our findings suggest that careful visualization and release of the distal TCL is equally important during OCTR. As incomplete distal TCL release is a known concern with ECTR, some surgeons use a dual-portal approach in hopes of preventing this issue, although existing literature has not demonstrated a difference in outcomes with this approach compared with single-portal ECTR.^30^ Regardless of the specific approach and strategies used, the proximal and distal releases of the TCL should be considered critical portions of the procedure deserving of particular care and attention by the surgeon.

We observed no difference in prevalence of nerve injury, the most serious complication of CTR, during revision surgery, in keeping with meta-analyses reporting no significant increase in rates of permanent nerve injury associated with ECTR.^1,18,19,31^ However, our study was not designed to examine the incidence of iatrogenic nerve injury during CTR, as we did not capture injuries recognized and managed intraoperatively nor those treated postoperatively without a concomitant revision CTR *CPT* code.

### Limitations

This study has several limitations. Intraoperative findings during revision surgery were based on surgeons’ documentation, which is inherently subjective and variable; therefore, results of our secondary analysis are intended for hypothesis generation and require confirmation in prospective studies. Our reliance primarily on billing codes to assign procedure laterality is predisposed to misclassification. Although manual electronic medical record review allowed us to confirm revision CTRs and reclassify false positives, we may have missed revisions with incorrectly coded laterality, leading to underestimation of revision CTR incidence (although not likely affecting revisions following ECTR vs OCTR differentially). We could not capture CTRs occurring outside the VHA, potentially further contributing to underestimation of revision CTR incidence. Despite our efforts to mitigate confounding in this observational study, the increased risk of revision associated with ECTR may have resulted from an unmeasured confounder, such as individual surgeon experience with the ECTR technique, which we were unable to account for directly. Additionally, distinct characteristics of the VHA population, such as male predominance and high prevalence of comorbidities, may impact the generalizability of our findings, although we endeavored to control for these factors in our statistical analysis to the extent possible.

## Conclusions

In this cohort study of revision CTR in the largest integrated health care system in the US, ECTR was associated with increased risk of revision compared with OCTR, but the absolute risk was low regardless of technique. Intraoperative findings at revision varied significantly according to index CTR technique.

## Notes

Supplement 1.
eMethods.
**eFigure.** Study Profile

Supplement 2.
Data Sharing Statement
