# Supplementary material for: Revision Carpal Tunnel Release Following Endoscopic Compared With Open Decompression
Source: JAMA Netw Open. 2024 Jan 12;7(1):e2352660. doi: 10.1001/jamanetworkopen.2023.52660 (PMC10787312; doi:10.1001/jamanetworkopen.2023.52660)
Supplement: Supplement 1. — eMethods. eFigure. Study Profile [file jamanetwopen-e2352660-s001.pdf]

# Supplemental Online Content

Ferrin PC, Sather BK, Krakauer K, Schweitzer TP, Lipira AB, Sood RF. Revision carpal tunnel release following endoscopic compared with open decompression. *JAMA Netw Open*. 2024;7(1):e2352660. doi:10.1001/jamanetworkopen.2023.52660

**eMethods.**

**eFigure.** Study Profile

This supplemental material has been provided by the authors to give readers additional information about their work.

© 2024 Ferrin PC et al. *JAMA Network Open.*

**Supplemental Methods: Determination of Procedure Laterality**

Of 135,920 carpal tunnel release (CTR) procedures performed for carpal tunnel syndrome on 104,399 patients, 124,088 (91.3%) had CPT modifier codes specifying laterality. In order to minimize the number of procedures excluded for missing laterality data, natural language processing of operative reports was applied to the remaining procedures, allowing for laterality to be assigned in 10,828 (91.5%) of the 11,832 procedures without CPT modifier data. The 1,004 procedures for which laterality could not be assigned in either manner were necessarily excluded along with 304 additional CTR procedures performed in those 925 patients, for a total of 1,308 (0.96%) procedures on 925 (0.89%) patients exclude from the analysis (eFigure 1).

Accuracy of laterality assignment was assessed via manual chart review of operative reports and peri-procedural clinical notes for 200 randomly selected procedures, sampling equally from open and endoscopic CTRs (100 each) and from procedures with laterality determined by CPT modifier codes and from natural language processing (100 each). Laterality was specified correctly in 199 of 200 cases (99.5%, 95% CI: 97.2-99.9%) overall. Accuracy did not differ significantly according to whether laterality was assigned by CPT modifiers (100/100 CTRs) or natural language processing (99/100 CTRs; $p = 1.00$, Fisher's exact test) nor by CTR technique, with laterality assigned correctly in 100/100 open compared to 99/100 endoscopic CTRs ($p = 1.00$, Fisher's exact test).

© 2024 Ferrin PC et al. *JAMA Network Open.*

**eFigure 1**. Study profile. CDW, Veterans Health Administration Corporate Data Warehouse; CTR, carpal tunnel release; CTS, carpal tunnel syndrome.

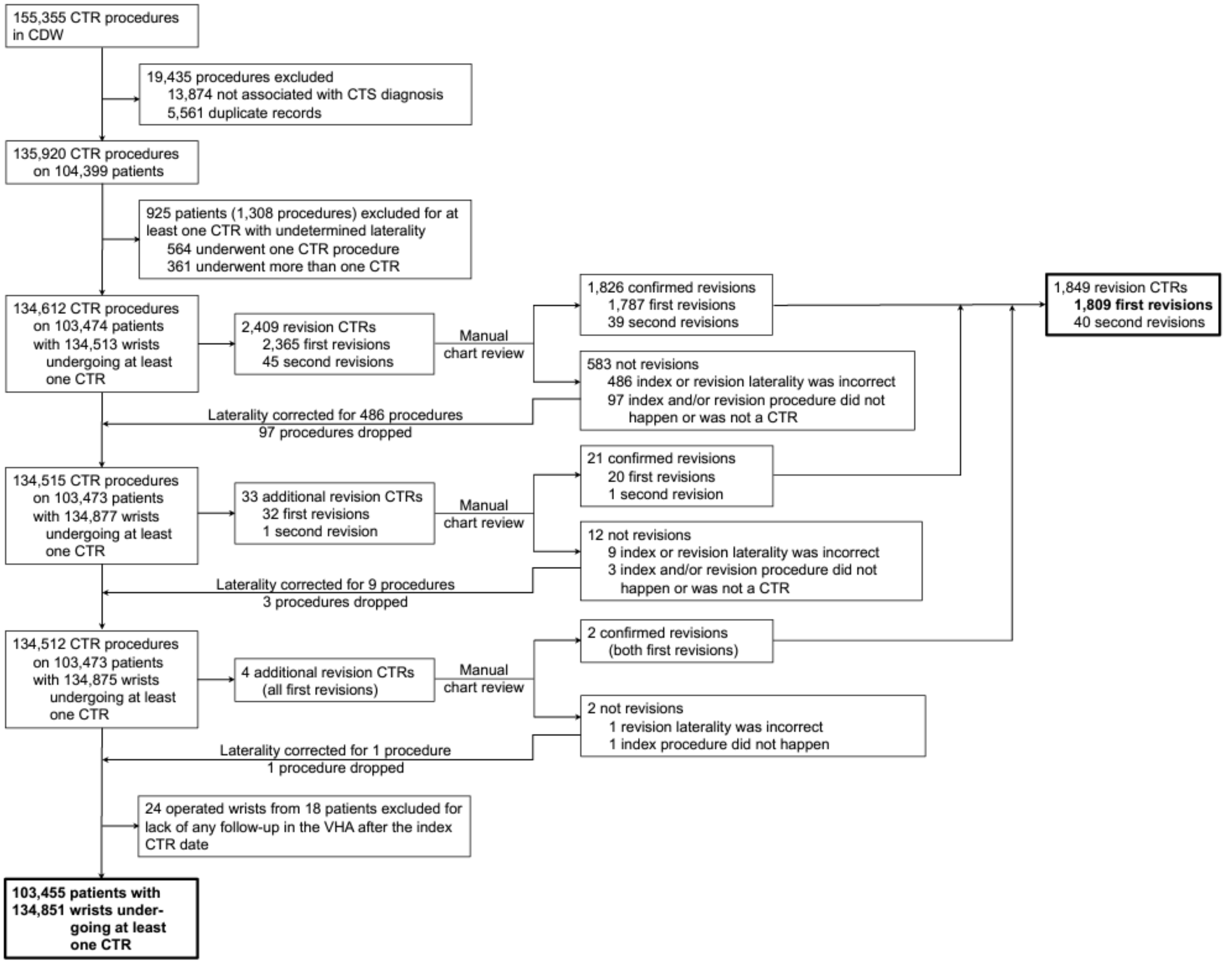


© 2024 Ferrin PC et al. *JAMA Network Open.*
