# Supplementary material for: Revision Carpal Tunnel Release Following Endoscopic Compared With Open Decompression
Source: JAMA Netw Open. 2024 Jan 12;7(1):e2352660. doi: 10.1001/jamanetworkopen.2023.52660 (PMC10787312; doi:10.1001/jamanetworkopen.2023.52660)
Supplement: Supplement 2. — Data Sharing Statement [file jamanetwopen-e2352660-s002.pdf]

Data Sharing Statement

Ferrin. Revision Carpal Tunnel Release Following Endoscopic Compared With Open Decompression. *JAMA Netw Open*. Published January 12, 2024.
doi:10.1001/jamanetworkopen.2023.52660

Data
Data available: No
